# The gyrfalcon (*Falco rusticolus*) genome

**DOI:** 10.1093/g3journal/jkad001

**Published:** 2023-01-05

**Authors:** Andrea Zuccolo, Sara Mfarrej, Mirko Celii, Saule Mussurova, Luis F Rivera, Victor Llaca, Nahed Mohammed, Arnab Pain, Abdulmajeed Fahad Alrefaei, Abdulwahed Fahad Alrefaei, Rod A Wing

**Affiliations:** Center for Desert Agriculture (CDA), Biological and Environmental Sciences & Engineering Division (BESE), King Abdullah University of Science and Technology (KAUST), Thuwal 23955-6900, Saudi Arabia; Crop Science Research Center, Sant’Anna School of Advanced Studies, Piazza Martiri della Libertà 33, 56127 Pisa, Italy; King Abdullah University of Science and Technology (KAUST), Pathogen Genomics Laboratory, Biological and Environmental Science and Engineering (BESE), Thuwal-Jeddah 23955-6900, Saudi Arabia; Center for Desert Agriculture (CDA), Biological and Environmental Sciences & Engineering Division (BESE), King Abdullah University of Science and Technology (KAUST), Thuwal 23955-6900, Saudi Arabia; Center for Desert Agriculture (CDA), Biological and Environmental Sciences & Engineering Division (BESE), King Abdullah University of Science and Technology (KAUST), Thuwal 23955-6900, Saudi Arabia; Center for Desert Agriculture (CDA), Biological and Environmental Sciences & Engineering Division (BESE), King Abdullah University of Science and Technology (KAUST), Thuwal 23955-6900, Saudi Arabia; Research and Development, Corteva Agriscience, Johnston, IA 50131, USA; Center for Desert Agriculture (CDA), Biological and Environmental Sciences & Engineering Division (BESE), King Abdullah University of Science and Technology (KAUST), Thuwal 23955-6900, Saudi Arabia; King Abdullah University of Science and Technology (KAUST), Pathogen Genomics Laboratory, Biological and Environmental Science and Engineering (BESE), Thuwal-Jeddah 23955-6900, Saudi Arabia; Department of Biology, Jamoum University College, Umm Al-Qura University, Mecca 24382, Saudi Arabia; Department of Zoology, College of Science, King Saud University, P.O. Box 2455, Riyadh 11451, Saudi Arabia; Center for Desert Agriculture (CDA), Biological and Environmental Sciences & Engineering Division (BESE), King Abdullah University of Science and Technology (KAUST), Thuwal 23955-6900, Saudi Arabia; School of Plant Sciences, Arizona Genomics Institute, University of Arizona, 24 Tucson, Arizona 85721, USA

**Keywords:** gyrfalcon, *Falco rusticolus*, conservation genomics, long reads, transposable elements, CR1, chromosome fusion

## Abstract

High-quality genome assemblies are characterized by high-sequence contiguity, completeness, and a low error rate, thus providing the basis for a wide array of studies focusing on natural species ecology, conservation, evolution, and population genomics. To provide this valuable resource for conservation projects and comparative genomics studies on gyrfalcon (*Falco rusticolus*), we sequenced and assembled the genome of this species using third-generation sequencing strategies and optical maps. Here, we describe a highly contiguous and complete genome assembly comprising 20 scaffolds and 13 contigs with a total size of 1.193 Gbp, including 8,064 complete Benchmarking Universal Single-Copy Orthologs (BUSCOs) of the total 8,338 BUSCO groups present in the library aves_odb10. Of these BUSCO genes, 96.7% were complete, 96.1% were present as a single copy, and 0.6% were duplicated. Furthermore, 0.8% of BUSCO genes were fragmented and 2.5% (210) were missing. A de novo search for transposable elements (TEs) identified 5,716 TEs that masked 7.61% of the *F. rusticolus* genome assembly when combined with publicly available TE collections. Long interspersed nuclear elements, in particular, the element Chicken-repeat 1 (CR1), were the most abundant TEs in the *F. rusticolus* genome. A de novo first-pass gene annotation was performed using 293,349 PacBio Iso-Seq transcripts and 496,195 transcripts derived from the assembly of 42,429,525 Illumina PE RNA-seq reads. In all, 19,602 putative genes, of which 59.31% were functionally characterized and associated with Gene Ontology terms, were annotated. A comparison of the gyrfalcon genome assembly with the publicly available assemblies of the domestic chicken (*Gallus gallus),* zebra finch (*Taeniopygia guttata*), and hummingbird (*Calypte anna*) revealed several genome rearrangements. In particular, nine putative chromosome fusions were identified in the gyrfalcon genome assembly compared with those in the *G. gallus* genome assembly. This genome assembly, its annotation for TEs and genes, and the comparative analyses presented, complement and strength the base of high-quality genome assemblies and associated resources available for comparative studies focusing on the evolution, ecology, and conservation of Aves.

## Introduction

**Fig. 1.**
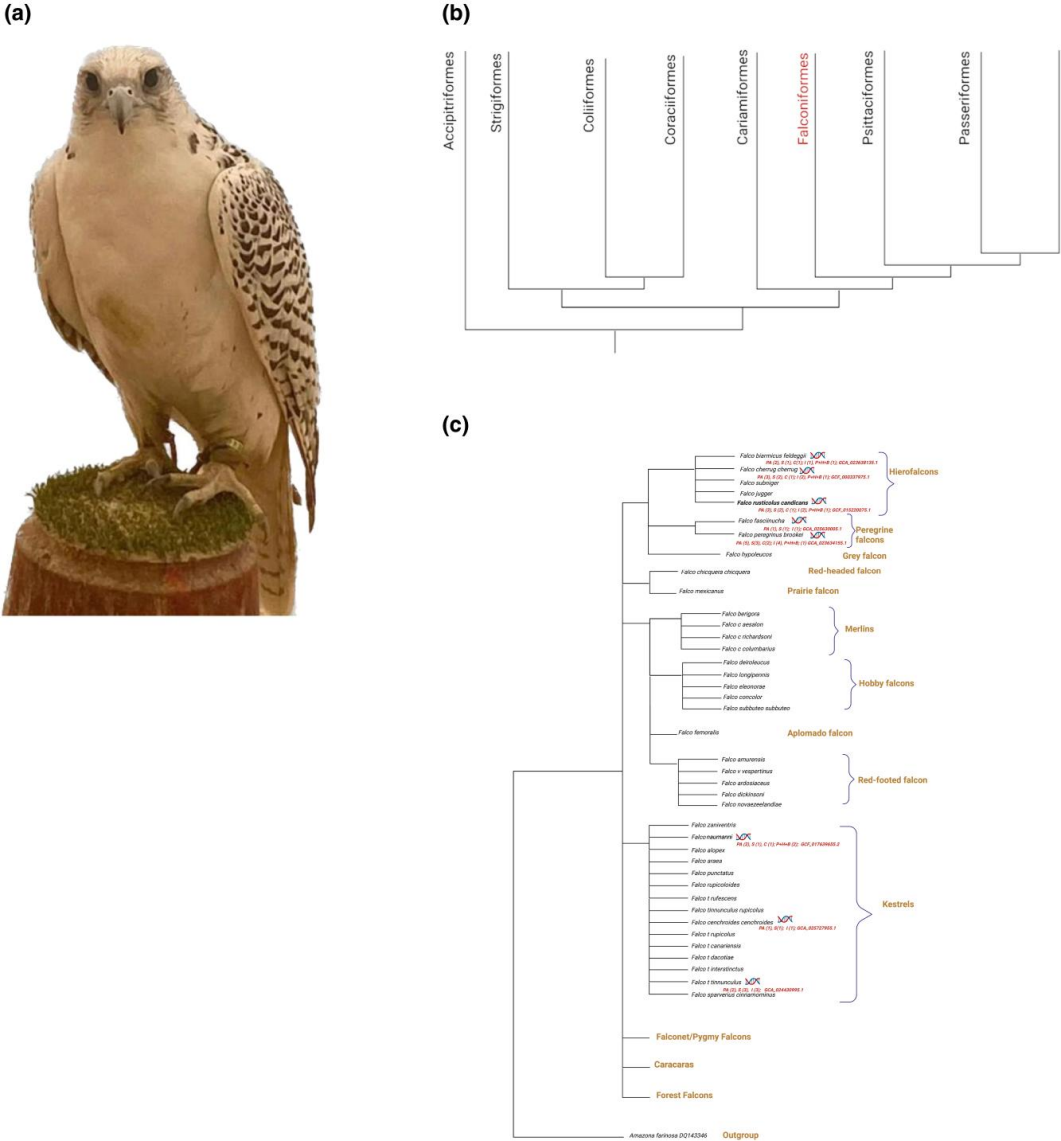
a) Picture of a gyrfalcon. b) Placement of falcons in the avian tree of life (modified and simplified from Prum et al., 2015 and Wink, 2018). c) Phylogenetic analysis of falcons modified and simplified from Wink (2018). For the species that have been sequenced (indicated by a double-helix) we provided along to the genome assembly accession number, the genome assembly details in parentheses are as follows: PA, number of primary assemblies; S, number of assemblies at the scaffold level; c, number of assemblies at the chromosome level; i, Illumina technology; p, PacBio technology; h, HiC chromatine interaction data; b, bionano optical maps. For *F. pelegrinoides* which was sequenced but is not included in the phylogenetic tree, the genome assembly information are PA (1), S (1), (i).

Of the extant tetrapod vertebrates, birds (Aves) are the most diverse lineage (Prum et al., 2015) and include at least 40 orders that comprise over 10,000 living species (Brusatte et al., 2015). These represent the extant members of an adaptive radiation period that occurred approximately 150 Ma (Chiappe and Dyke, 2002). Aves are characterized by a high diversity in morphology, ecology, and behavior (Gill, 1995). The genus to which falcons belong is a part of the Falconinae subfamily of the family Falconidae and comprises 38 species that are widely distributed throughout Asia, North America, and Europe (Wink, 2018) (Fig. 1). Falcons can be roughly categorized into the following four groups: Kestrels, Hierofalcons, Peregrine falcons, and Hobbies (Wink, 2018).

The genus *Falco* underwent rapid and recent diversification, and evolutionary studies have investigated the ecological and geological factors driving it. The divergence within the subfamily Falconinae was inferred to date back to approximately 16 My, and the most species-rich genus, *Falco*, which comprises about 60% of all Falconidae species, began to diverge approximately 7.5 Ma (Fuchs et al., 2015). The timescale over which the falcons diverged and diversified is comparable to that of early hominids (Cade and Digby, 1982). Falcons underwent several radiations, which led to a higher diversity than that of most genera of Aves (Gill and Donkser, 2019). The unique evolutionary history of the genus *Falco* offers the possibility to study its speciation mechanisms at different evolutionary stages. Advances in genomics allow a better molecular-level understanding of the evolutionary mechanisms involved in generating the large diversity of falcons by investigating their genome sequence, structure, and function.

Most bird species have diploid karyotypes containing approximately 80 chromosomes. Generally, they comprise 7–10 pairs of large- and medium-sized chromosomes (macrochromosomes), several microchromosomes (30–33 pairs), often morphologically indistinguishable and the sex chromosomes (Masabanda et al., 2004). The karyotype of the Falconidae of the order Falconiformes is markedly different from this general pattern. The chromosome number per diploid genome is low and ranges from 40 chromosomes in merlin (*Falco columbarius*) to 52 in common kestrel (*Falco tinnunculus*). Additionally, the macrochromosomes show little size difference, and the number of microchromosomes is low. The fusion of microchromosomes with macrochromosomes is the likely mechanism that leads to low chromosome counts in falcons. Indeed, tandem fusions of microchromosomes with macrochromosomes and those between microchromosomes have frequently been observed (Nishida et al., 2008).

The gyrfalcon (*Falco rusticolus*) is the largest and one of the fastest flying falcon. In both sexes, the average body length ranges from 41 to 56 cm, and the average body weight is between 800 and 2,100 g (Fig. 1). The species is polymorphic; hence, its plumage varies greatly in color according to the environment in which it lives; it can have white, black, brown, or dark brown feathers (del Hoyo-Andre and Cabot, 1994).

Substantial advances in sequencing technology coupled with efficient assembly strategies and the availability of long-range sequencing technologies, such as optical mapping and HiC chromatin interaction-based analyses, have dramatically increased the overall quality of genome assemblies (Sedlazeck et al., 2018). Such high-quality genome assemblies constitute valuable resources for any thorough investigation of wild and domesticated species ecology, conservation, evolution, and population genetics (Whibley et al., 2021). To provide genomic resources for comparative and conservation projects, we built an accurate and complete genome sequence of gyrfalcon using third-generation sequencing strategies with the support of optical maps. We obtained a genome assembly characterized by high contiguity and completeness, including 33 scaffolds and contigs of a total size of 1.193 Gbp. The Benchmarking Universal Single-Copy Orthologs (BUSCO) analysis identified 8,064 complete BUSCOs of the 8,338 Aves BUSCO groups, corresponding to 96.7% complete and 0.8% fragmented genes. The genome assembly was searched de novo for transposable elements (TEs), resulting in the identification of 5,716 TEs. Together with publicly available resources, these masked 7.61% of the entire genome assembly. Furthermore, 19,602 putative genes resulted from a first-pass annotation with the software Augustus (Stanke and Waack, 2003) using 293,349 PacBio Iso-Seq transcripts and 496,195 transcripts derived from the assembly of 42,429,525 Illumina paired-end RNA-seq reads as extrinsic support. Whole-genome comparisons with publicly available genomes of the domestic chicken (*Gallus gallus,*Warren et al., 2017), zebra finch (*Taeniopygia guttata,*Warren et al., 2010), and hummingbird (*Calypte anna*, Rhie et al., 2021) revealed several genome rearrangements encompassing all classes of structural variants that included nine chromosome fusions compared with the *G. gallus* genome.

This genome assembly adds to those already available for birds (Bravo et al., 2021), specifically, falcons (Zhan et al., 2013; Doyle et al., 2018; Cho et al., 2019; Wilcox et al., 2022) (Fig. 1), complementing and strengthening the base of high-quality genome assemblies for comparative studies focusing on the evolution, ecology, and conservation of Aves.

## Materials and methods

### DNA extraction

High molecular weight DNA was extracted from the blood of an Arabian 2-year-old female gyrfalcon weighing 1,400 g using the Nanobind magnetic disc-based method from Circulomics (Circulomics, Baltimore, MD, USA). The optimization included efficient homogenization of the blood with phosphate-buffered saline (PBS) to avoid a viscous lysate and ensure proper lysis, considering avian erythrocytes contain high amounts of nuclei. 50 μL of avian blood provided more than 20 μg of DNA yield, quantified using broad range Qubit fluorometer (Invitrogen, USA). The DNA fragment size was 120 kb on the pulsed field gel electrophoresis.

### RNA extraction

RNA was extracted from falcon blood using Zymo-Direct Zol kit (Zymobiomics, Zymo Research Corporation, Irvine, CA, USA) with DNase I treatment. To get a clean aqueous RNA layer, the TRIzol (Invitrogen, USA) lysis incubation was extended, and chloroform was added during the initial lysis step. The quality control of purified RNA was performed using broad range Qubit kit (Invitrogen, USA) and RNA 6000 Nano LabChip kit (Agilent, Santa Clara, CA, USA) respectively, with an RNA integrity number of 9.

### PacBio Libraries construction

PacBio DNA read and RNA Iso-Seq libraries were constructed by the KAUST Bioscience Core Lab using standard PacBio protocols. The cDNA synthesis was done using NEBNext Single Cell/Low input cDNA synthesis and amplification module (NEB, Cat No.: E6421S) with Iso-Seq Express Oligo Kit (Pacbio, 101-737-500) and Iso-seq library was built with SMRTbell Express Template Prep Kit 2.0 (PacBio, 100-938-900).

### DNA Sequencing

HMW gDNA was sequenced using the PacBio single-molecule real-time (SMRT) platform. Two SMRT cells were run for 15 h per cell with the continuous long read (CLR) method generating 13.93 million reads for a total of 345.3 Gbp of sequence data and providing an estimated ∼287.8× coverage of the *F. rusticolus* genome, assuming a genome size of 1.2 Gbp (Wilcox, 2019).

### Assembly

Raw sequence data were assembled using two assemblers: MECAT2 (Xiao et al., 2017) and CANU v.1.8 (Koren et al., 2017). Both were run using default settings, assuming a genome size of 1.2 Gbp for the gyrfalcon genome.

### Polishing

Both the CANU and MECAT2 assemblies were subjected to two polishing rounds: First, PacBio reads were mapped onto each assembly using the software Blasr (Chaisson and Tesler, 2012). Subsequently, the software Arrow was used for polishing, as implemented in SMRTlink v.9.0 (Pacific Bioscience, Menlo Park, CA, USA) with default settings. Next, 412.7 million paired-end Illumina reads (151 bp/end) using DNA obtained from the same gyrfalcon blood sample were mapped onto both assemblies using bwa-mem (Li, 2013), followed by polishing with the software Pilon (Walker et al., 2014) under default settings.

### Quality evaluation

The initial assessment of the genome assembly metrics was conducted using the software Quast (Gurevich et al., 2013).

### Bionano library and hybrid scaffolding

Bionano optical maps was generated by Corteva Agriscience (Johnston, IA, USA). The genome assembly and the corresponding Bionano maps were employed to build a hybrid scaffold using the software Bionano solve v.3.4 (https://bionanogenomics.com/) with the following settings: -B 2 -N 2 -f. Data visualization was performed using Bionano Access (v12.5.0) software package.

### Iso-Seq data

RNA was sequenced using the PacBio SMRT platform using Sequel® II Binding Kit 2.1(Pacbio, 101-843-000), Sequel II Sequencing Kit 2.0 (101-820-200), and SMRT cell 8 M Tray (101-389-001). Full-length isoforms were extracted using the tools included in SMRTlink v.9.0 (Pacific Bioscience) to perform the following analyses: CCS extraction from CLR transcriptome reads using the tool CCS (v.4.2.0); barcode demultiplexing using the tool lima (v.1.11.0); removal of polyA and concatemers from full-length reads using the software isoseq3 refine (v.3.3.0) and clustering sequences using isoseq3 cluster software (v.3.3.0). Only the high-quality reads obtained through this pipeline were used for subsequent analyses. Iso-Seq reads were mapped onto the genome assembly using the tool minimap2 (Li, 2018) with the setting -ax splice:hq.

### RNA-seq

Paired-end RNA reads (42,429,525 151 bp) were assembled using the software Trynity v.2.8.5 (Grabherr et al., 2011). Predicted transcripts were then clustered using the software cd-hit (Li and Godzik, 2006) with the following settings: -c 0.98 -p 1 -d 0 -b 3.

### Comparison with the other bird genome-scale assemblies

The gyrfalcon genome assembly was compared with those of the domestic chicken (GenBank assembly accession: GCA_000002315.5), hummingbird (GenBank assembly accession: GCA_003957575.1), and zebra finch (GCA_000151805.2) using the software Mashmap (Jain *et al*., 2018) and D-genies (Cabanettes and Klopp, 2018).

### BUSCO assessment

BUSCO evaluations were performed for the gyrfalcon genome assembly using the BUSCO (v.5.1.2) software package (Simão et al., 2015) and the conserved gene library aves_odb10.2019-11-20.

### Circa plots

Circa plots were constructed using the Circa software available at https://omgenomics.com/circa/.

### Gene prediction and functional annotation

Gene prediction was performed using the software Augustus (Stanke and Waack, 2003), as implemented in the tool OmicsBox (BioBam, 2019), with gene models calculated for the domestic chicken and the Iso-Seq reads described above as extrinsic support. Functional annotation was performed, considering the best match for each transcript calculated using Diamond BLASTp (v.0.9) searches of predicted proteins against the nonredundant NCBI protein database. An e-value of 1e-6 was used as the threshold for significant matches. Gene ontology analysis was performed using InterProScan v.5.39 with default settings (Jones et al., 2014).

### TE identification and quantification

Using default settings, a TE library was obtained by running the software EDTA (Ou et al., 2018) on the gyrfalcon genome assembly. The RepBase (Bao et al., 2015) vertebrate TE library (July 2020) was combined with EDTA-predicted TEs to generate the de novo FALCON_TE_LIBRARY_V3.fa. Quantification of TEs in the *F. rusticolus* genome was performed with RepeatMasker software (Smit et al., 2015) using the FALCON_TE_LIBRARY_V3.fa with default settings, except for the fact that the option -qq was used to hasten the search.

## Results and discussion

### Genome assembly and statistics

The gyrfalcon genome was sequenced using two smart cells with PacBio Sequel sequencing technology. A total of 14,329,526 reads were produced, amounting to 351.8 Gbp of sequence data. Assuming an estimated genome size of ∼1.2 Gbp (Wilcox et al., 2019), the sequence data provided ∼293× genome coverage. The PacBio reads were used as inputs for two different genome assemblers, MECAT2 (Xiao et al., 2017) and CANU (Koren et al., 2017), followed by two rounds of polishing using the Illumina data and the PacBio reads (see Methods). The N50 and L50 values of contigs were 48.17 Mbp and 9 for CANU and 39.78 Mbp and 10 for MECAT2, respectively (Table 1). We used CANU for the baseline falcon genome assembly based on these metrics.

**Table 1.** Gyrfalcon genome assembly metrics.

	MECAT2	CANU
# contigs	1,161	1,219
# contigs > 50,000 bp	539	938
Total length (bp)	1,250,464,509	1,308,989,646
Largest contig (bp)	104,620,499	122,222,403
GC (%)	42.55	42.83
N50 (bp)	39,771,069	48,172,526
N75 (bp)	29,809,375	29,473,197
L50	10	9
L75	18	19
Ns per 100 kbp	0	0

### Construction and quality assessment of the edited hybrid genome assembly

The baseline genome assembly was then used with Bionano optical maps to produce a hybrid assembly using the Bionano solve software (www.bionanogenomics.com). The hybrid assembly comprised 70 super-scaffolds manually inspected for possible inconsistencies with the optical map. Conflicts were resolved by “breaking” the questionable super-scaffolds when necessary. This process resulted in a dataset consisting of 20 super-scaffolds and 13 contigs. Altogether, these 33 contigs and super-scaffolds represent the genome assembly of gyrfalcon (Table 2). These were numbered according to length, from the longest (122.3 Mbp) to the shortest (1.071 Mbp). The entire genome assembly has six gaps ranging from 183 to 113,856 bp. The total length of these gaps is 384,827 bp. The full length of the genome assembly is 1.193 Gbp, which is highly consistent with the estimated genome size of 1.2 Gbp for gyrfalcon (Wilcox et al., 2022).

**Table 2.** Gyrfalcon hybrid-scaffolded assembly description and similarity to other reference genomes.

Falcon assembly super-scaffold (sc) and contigs (co)	Length (bp)	Number of predicted genes	Genes (Mbp)	TE content	GC content	Gaps (Ns)	Chicken (chr)	Hummingbird (chr)	Zebra finch (chr)
1_sc	122,326,263	1,254	10.25	9.34	40.26	—	1	1	1
2_sc	104,580,349	1,945	18.60	5.39	42.78	1 (30,710)	12,14,28,2	12,14,28,2	12,14,28, 2
3_sc	104,319,969	974	9.34	9.18	40.31	—	2	2	2
4_sc	95,117,127	1,302	13.69	6.52	40.88	—	15,4	4A, 4B	15,4
5_sc	91,589,740	1,013	11.06	8.19	40.62	—	3	3	3
6_co	77,507,961	1,298	16.75	6.89	41.71	—	1	1	1,1A
7_sc	73,427,893	1,271	17.31	5.87	42.34	—	10,5	10,5A	10,5
8_sc	66,236,224	1,095	16.53	4.82	42.68	1 (113,856)	7,13	7,13	7, 13
9_sc	58,459,340	690	11.80	10.50	40.66	—	Z	Z	Z
10_sc	55,126,675	1,031	18.70	5.03	43.42	1 (33,710)	6,17	6,17	6,17
11_sc	37,966,174	925	24.36	4.60	43.87	—	20,5	20,5	20,5
12_co	35,775,413	671	18.76	4.87	42.25	—	8	8	24,8
13_sc	34,096,687	511	14.99	4.55	42.15	—	3	3	3
14_co	31,933,560	1,050	32.88	3.92	46.58	—	19,15,18	19,18,15	19,18,15
15_co	29,744,985	559	18.79	4.40	42.81	—	9	9	9
16_sc	27,799,139	302	10.86	11.16	40.34	1 (106,772)	Z	Z	Z
17_sc	24,209,053	456	18.84	5.43	43.51	—	4	Chr 4	4A
18_sc	23,548,544	485	20.60	4.08	42.55	—	11	11	11
19_sc	17,715,289	635	35.84	3.85	48.58	—	21,23	21,23	21, 23
20_co	14,691,556	148	10.07	9.43	39.62	—	1	1	1A
21_sc	12,341,734	106	8.59	46.03	41.43	1 (99,596)	Z	Z, W	Z
22_co	8,268,704	221	26.73	3.87	48.74	—	24	24	24
23_sc	8,146,813	192	23.57	5.63	44.2	—	12	12	12
24_co	6,660,032	343	51.50	6.40	52.48	—	27	27	27
25_sc	6,398,898	251	39.23	4.57	49.48	—	22	22	22
26_sc	5,459,845	104	19.05	33.03	43.12	1 (183)	Z	W, Z	?
27_co	5,078,416	207	40.76	3.05	50.27	—	26	26	26
28_co	4,212,995	350	83.08	5.50	57.69	—	25,33	25,33	25
29_sc	3,107,971	22	7.08	50.36	41.59	—	Z	Z	Z
30_co	2,469,339	130	52.65	4.06	53.38	—	26	26	26
31_co	2,304,856	29	12.58	55.54	41.8	—	?	W,Z	Z?
32_co	2,015,512	21	10.42	35.13	45.32	—	Z	Z, W,4B	Z
33_co	1,071,326	11	10.27	59.98	40.79	—	Z	Z?	Z?
TOTAL	1,193,708,382	19,602	16.42	7.61	42.13	6 (384,827)	—	—	—

To assess the completeness of our final assembly, we conducted a BUSCO analysis identifying 8,064 complete genes out of the 8,338 included in aves_odb10, which corresponds to 96.7% of the complete genes. Of these 96.1% were single copies, and 0.6% were duplicated. There were 0.8% fragmented and 210 (2.5%) missing genes.

The hybrid assembly included another 42 small super-scaffolds shorter than 1.8 Mbp, totaling 11.5 Mbp, which did not show any significant similarity with the genome of *G. gallus*. These short super-scaffolds could include unassigned microchromosomes and unique and highly divergent regions of the gyrfalcon genome or could be artifactual products of the hybrid scaffolding process generated mainly because of highly repetitive simple sequences.

### Comparative genomics with domestic chicken, zebra finch, and hummingbird

The entire *F. rusticolus* genome assembly was compared with the complete high-quality genome assemblies of the domestic chicken (*G. gallus*), zebra finch (*T. guttata*), and hummingbird (*C. anna*). For all 33 contigs and super-scaffolds included in the assembly, we were able to find solid indication of homology to at least one chromosome of the chicken, zebra finch, and hummingbird.

Eight gyrfalcon contigs/scaffolds exhibited similarity to W and/or Z sex chromosomes of one or more of the three species compared. To disentangle which of the gyrfalcon contigs/scaffolds were W or Z, we leveraged the known differential TE content across bird sex chromosomes (Peona et al., 2021). The W chromosome in birds possesses a higher TE content than the other chromosomes (Z chromosome included). Indeed, the TE content of the W chromosome is often >50%, whereas that of the other chromosomes tends to be <10%. Our results showed (Table 2) that *F. rusticolus* contigs 31, 32, and 33 and scaffolds 21, 26, and 29 had TE contents higher than 33% (Table 2), therefore they were assigned to the W sex chromosome, and the remaining two scaffolds to the Z chromosome.

### Comparisons with the domestic chicken genome: identification of chromosomal rearrangements

Comparisons between the *F. rusticolus* genome and the other three high-quality bird reference genomes showed a remarkable level of conservation in terms of sequence synteny and overall sequence similarity. Long stretches of contiguous sequences with an overall sequence similarity of >85% were observed (Supplementary Figs. S1–S3). This high overall level of conservation among the four species is remarkable, considering the fact that they diverged >60 Ma (Prum et al., 2015; Cho et al., 2019).

**Fig. 2.**
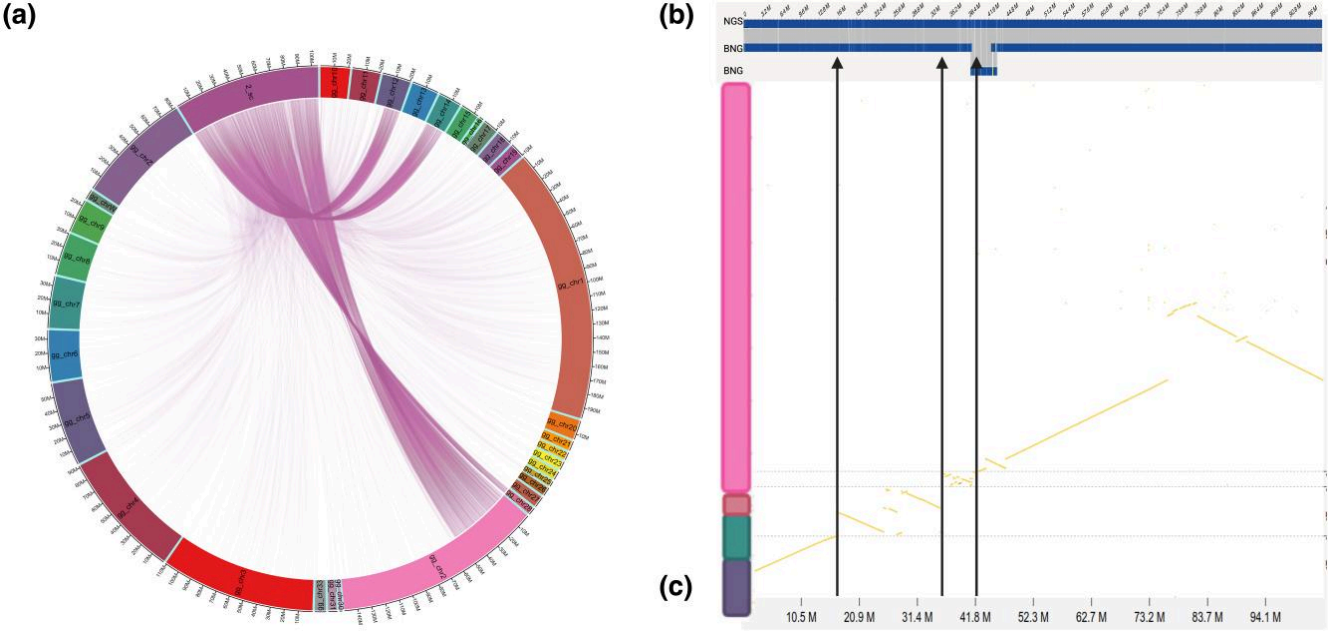
Details of predicted chromosomal rearrangements in the gyrfalcon super-scaffold 2_sc. a) Circa plot comparing 2_sc with the entire set of domestic chicken chromosomes (specified as “gg_chromosome number”). Regions showing significant similarity are connected by violet lines. b) Bionano optical map validation for 2_sc. NGS, assembled sequence; BNG, Bionano map. c) Dot plot of 2_sc vs four chicken chromosomes showing homology. 2_sc is on the *x*-axis, and chicken chromosomes are on the *y*-axis. The chicken chromosomes are coded using the color assigned to them in A.

However, major chromosomal rearrangements, including structural variants such as inversions and translocations, were identified, along with evidence of chromosomal fusions and breakages. Particularly, 9 of the 33 falcon genome assembly contigs and super-scaffolds appeared to result from chromosome fusions compared with the chicken genome (Table 2). Seven contigs demonstrated homology to two chicken chromosomes each, one to three chicken chromosomes, and one (2_sc, whose total length is 104.5 Mbp) included regions homologous to tracts from four different chicken chromosomes (Fig. 2). Tracts of the super-scaffold 2_sc from 16.3 to 35.9 Mbp and from 35.9 to 42.2 Mbp showed similarity to the entire length of chicken chromosomes 14 and 28, respectively. Two other regions were homologous to extensive stretches of chicken chromosome 12 (from the beginning of 2_sc to 16.3 Mbp) and chromosome 2 (from 42.2 Mbp to the end of 2_sc). All genome assembly regions in which potential rearrangements were detected were independently validated with optical maps, thereby eliminating any possible misassembled artifacts (Supplementary Figs. S4–S11).

The large number of genome rearrangements in the gyrfalcon genome was consistent with the evidence collected in other species of the *Falcon* genus, such as *Falco peregrinus* (Nishida et al., 2008; O’Connor et al., 2018; Penalba et al., 2020), indicating that the Falconiformes karyotype is an exception to the avian karyotypes otherwise characterized by a limited number of interchromosomal changes (Damas et al., 2018).

Of note, five contigs (24_co, 25_co, 27_co, 28_co, 30_co) which are not part of sex chromosomes showed features typical of microchromosomes such as high GC content, high gene density and TE depletion (McQueen et al., 1998; Warren et al., 2017; Waters et al., 2021). Specifically, they had a length ranging from 2.47 to 6.06 Mbp, a GC content (50.27–57.69%) significantly higher than the average one (42.83%), a TE content (3.05 to 6.40%) lower than average (7.61%) and a gene density (40.76–83.30 genes/Mbp) strikingly higher than the average one, i.e. 16.4 genes/Mbp (Table 2).

Interestingly, the comparative analysis of 28_co (Supplementary Fig. S5) showed a convincing similarity of this sequence to the microchromosomes 25 and 33 of *G. gallus* providing a suggestive example of microchormosomes fusion leading to chromosome reduction number as described in Joseph et al., 2018. These kind of rearrangements involving microchromosomes are not frequent in birds with the notable exceptions of Falconiformes and Psittaciformes (Joseph et al., 2018; O’Connor et al., 2018; Kretschemer et al., 2021).

### TE identification, abundance, and distribution

Using the EDTA pipeline (Ou et al., 2019), a library of TEs was created for *F. rusticolus*. This library included 5,716 entries (Supplementary file S2) and was used together with the RepBase (Bao et al., 2015) vertebrate TE library (5,038 entries) to mask the falcon genome assembly. Altogether, the TE library included 10,759 entries (Supplementary Table S1) and repeat-masked 7.61% of the genome assembly (Table 3).

**Table 3.** Gyrfalcon transposable element content.

TE class	Count	bp Masked	%masked
hAT	4,405	906,713	0.07%
CACTA	72,610	16,829,073	1.41%
Harbinger	7,017	1,206,649	0.10%
Mutator	29,652	6,480,296	0.54%
Mariner	8,693	1,510,257	0.13%
Helitron	5,461	3,135,059	0.26%
Other DNA TE	9,200	814,246	0.07%
LINE	57,441	29,573,603	2.66%
LTR-RT Copia	146	58,429	0.00%
LTR-RT-Gypsy	15,529	8,696,215	0.73%
LTR-RT	19,187	9,510,118	0.80%
SINE	3,986	524,148	0.04%
Others	37,396	11,572,395	0.80%
Total	270,723	90,817,201	7.61%

**Fig. 3.**
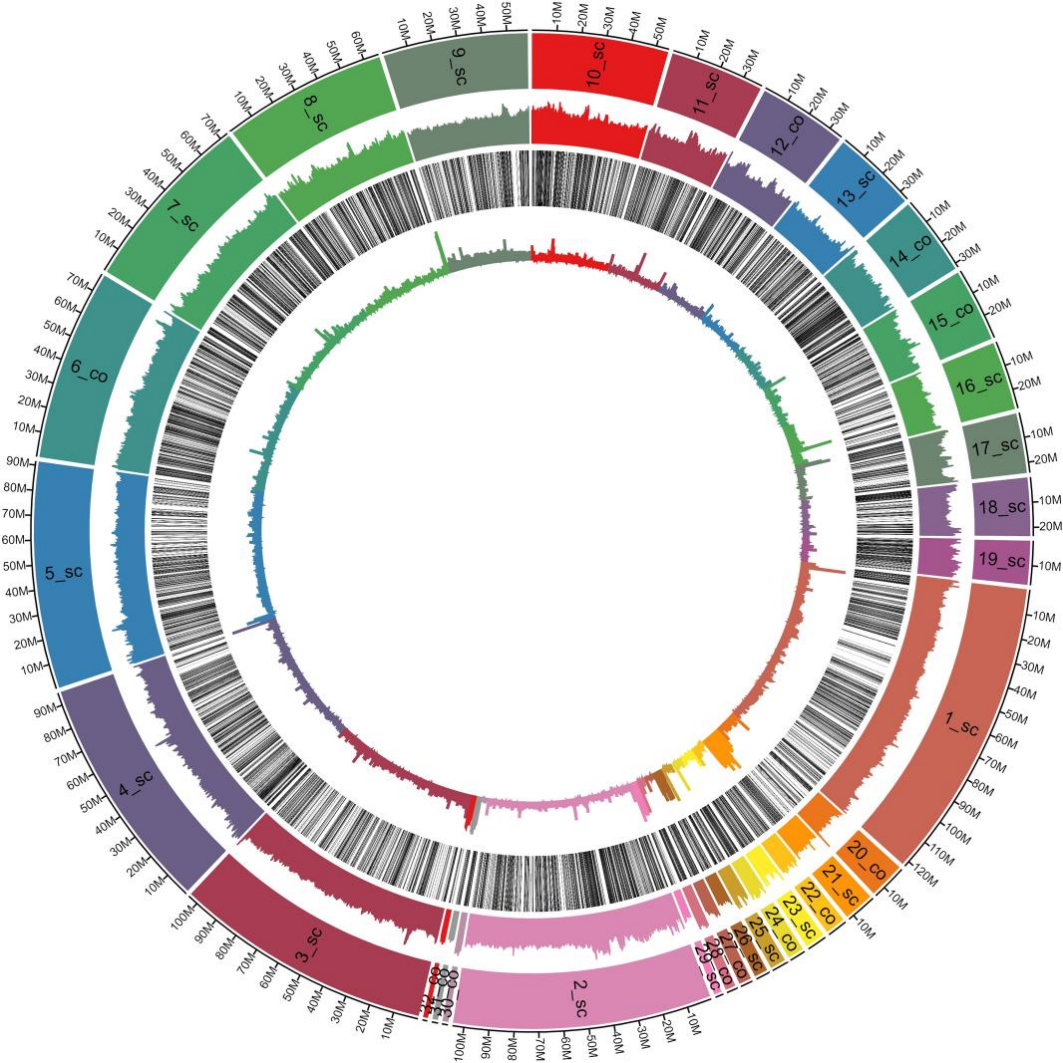
Circa plot of the *Falco rusticolus* genome assembly showing (outer circle inward) GC content distribution, gene density, and TE content.

The overall amount of TEs in the falcon genome was similar to that observed in the genomes of other birds and confirmed the underrepresentation of TEs in these genomes compared with those in the other metazoans. Notably, the overall number of TEs is higher than that estimated in previous studies on falcon species. Zhan *et al*., in 2013, estimated the amount of the repetitive fraction (not limited to TEs) in both saker and peregrine falcons to be 6.80%. Zhang *et al*., in 2014, estimated the TE content of *F. peregrinus* to be 5.50%. Recently, Wilcox *et al*. (2022) annotated an average of 6.5% of the genome of eight *Falcon* species as TE-related. However, the amount of TEs was highly variable across the different scaffolds and contigs and ranged from 3.05 to 59.98% (Table 2 and Fig. 3). For example, three contigs/scaffolds tentatively assigned to chromosome W had a TE content of >50%. The abundance of different TE types was consistent with that observed in other birds; all the main TE classes were represented, with LINEs being the most abundant (2.66%).

Most LINEs in the gyrfalcon assembly exhibited high similarity with the element “Chicken-repeat 1” (CR1), an element highly abundant in most published bird genomes. CR1 retrotransposons are the most common family of TEs found in the genomes of birds, crocodilians, turtles, and snakes (Suh *et al*., 2014). The length of the complete CR1 element is approximately 4.5 kbp; however, most copies are incomplete. Indeed, of all the CR1 paralogs in the chicken, only 0.6% are complete (International Chicken Genome Sequencing Consortium, 2004). Complete CR1 elements encode for two proteins: an RNA-binding protein (ORF1p) and a multifunctional protein with endonuclease and reverse transcriptase enzymatic activities (ORF2p). A TblastN search of the gyrfalcon genome, using a 100-amino acid tract of the CR1 ORF2 as a query, showed 13,124 significant (e-value 1e-5) hits. Theoretically, if all these hits corresponded to complete full-length elements, it would translate to 59.1 Mbp of sequence, amounting to ∼5% of the genome assembly. This is not the case in our assembly because the entire LINE complement totaled 2.66% of the assembly, thereby indicating the presence of many incomplete CR1 elements in the gyrfalcon genome.

### Gene annotation

*De novo* gene identification analysis was performed on the falcon genome assembly masked for TEs. The search used the gene predictor Augustus (Stanke and Waack, 2003) in the OmicsBox (BioBam, 2019) software package. As extrinsic support data, 293,349 RNA high-quality PacBio Iso-Seq sequences and 496,195 transcripts derived from the assembly of 42,429,525 Illumina PE RNA-seq reads were used. The Hidden Markov Model (HMM) used for the ab initio search was that computed for *G. gallus*. The search identified 19,602 putative coding regions (Fig. 3), 2,069 of which appeared to be monoexonic. This gene count is on par with the values obtained for other falcons, such as *F. peregrines* (16,263 genes) and *Falco cherrug* (16,204) (Zhan et al., 2013) and is consistent with the number of genes in many bird genomes (Zhang et al., 2014).

The average gyrfalcon gene length was 20.8 kbp, with a median of six exons per gene and an average of eight exons per gene. The overall number of introns was 146,180, with an average length of 2,606 bp. Of the predicted genes, 17,084 (87.15%) had InterProScan positive hits and 10,625 (59.31%) could be functionally characterized and associated with Gene Ontology terms. A description of the locations and structures of the predicted genes is available in the Supplementary file S1. As further support to the genome assembly completeness, it is worth noting that 291,029 Iso-Seq reads of the 293,249 (99.25%) reads were mapped onto the gyrfalcon assembly.

## Supplementary Material

jkad001_Supplementary_Data

## Data Availability

The genomics data presented in this manuscript were submitted to the National Center for Biotechnology Information with the BioProject accession number BioProject ID PRJNA872351. The genome assembly project has been deposited at DDBJ/ENA/GenBank under the accession JAPSEQ000000000. The version described in this paper is version JAPSEQ010000000. Supplementary file S1 and Supplementary file S2 are available in figshare: https://doi.org/10.25387/g3.21769628. Supplemental material available at G3 online.
