# Supplementary material for: The gyrfalcon (Falco rusticolus) genome
Source: G3 (Bethesda). 2023 Jan 5;13(3):jkad001. doi: 10.1093/g3journal/jkad001 (PMC9997569; doi:10.1093/g3journal/jkad001)
Supplement: jkad001_Supplementary_Data [file jkad001_supplementary_data.zip › Supplemental_Table_1_G3-2022-403823.docx]

Supplemental Table 1. TE content of gyrfalcon library

| TE class | EDTA | RepBase | Total |
| --- | --- | --- | --- |
| DTC (CACTA) | 3,301 | - | 3,301 |
| DTM (Mutator) | 1,267 | - | 1,267 |
| DTH (hAT) | 263 | 613 | 876 |
| DTA (Harbinger) | 159 | 198 | 357 |
| DTT (Mariner) | 202 | 293 | 495 |
| Uncharacterized DNA-TE | - | 439 | 439 |
| LTR-RT | 277 | 1,221 | 1,498 |
| Helitron | 245 | 48 | 293 |
| ERV | - | 776 | 776 |
| DIRS | - | 184 | 184 |
| LINE | - | 807 | 807 |
| Penelope | - | 93 | 93 |
| SINEs | - | 120 | 120 |
| Others | - | 251 | 251 |
