# Supplementary figures and images for: The gyrfalcon (Falco rusticolus) genome
Source: G3 (Bethesda). 2023 Jan 5;13(3):jkad001. doi: 10.1093/g3journal/jkad001 (PMC9997569; doi:10.1093/g3journal/jkad001)

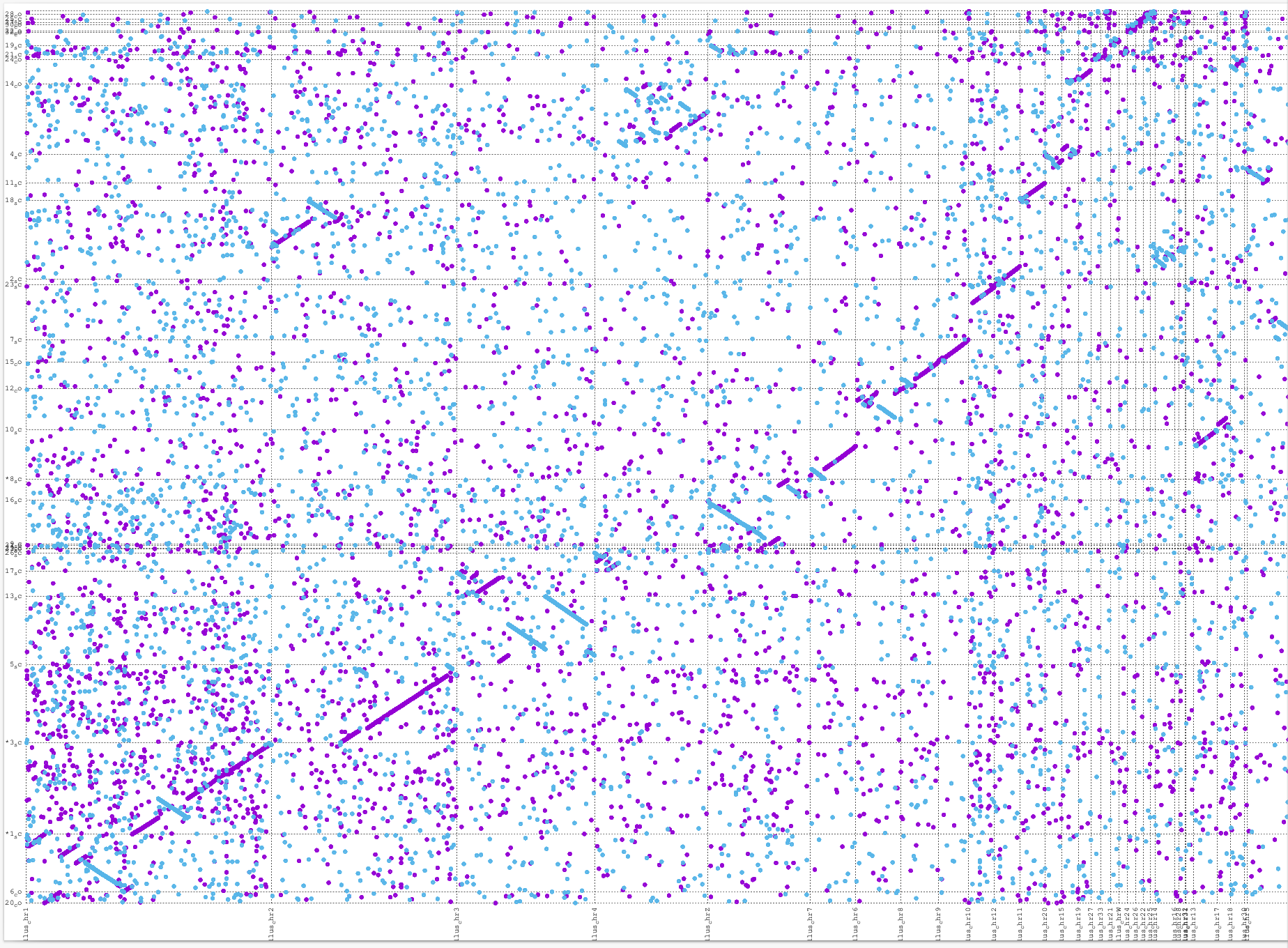

Supplement: jkad001_Supplementary_Data [file jkad001_supplementary_data.zip › Supplemental_Figure_1_G3-2022-403823.tif]

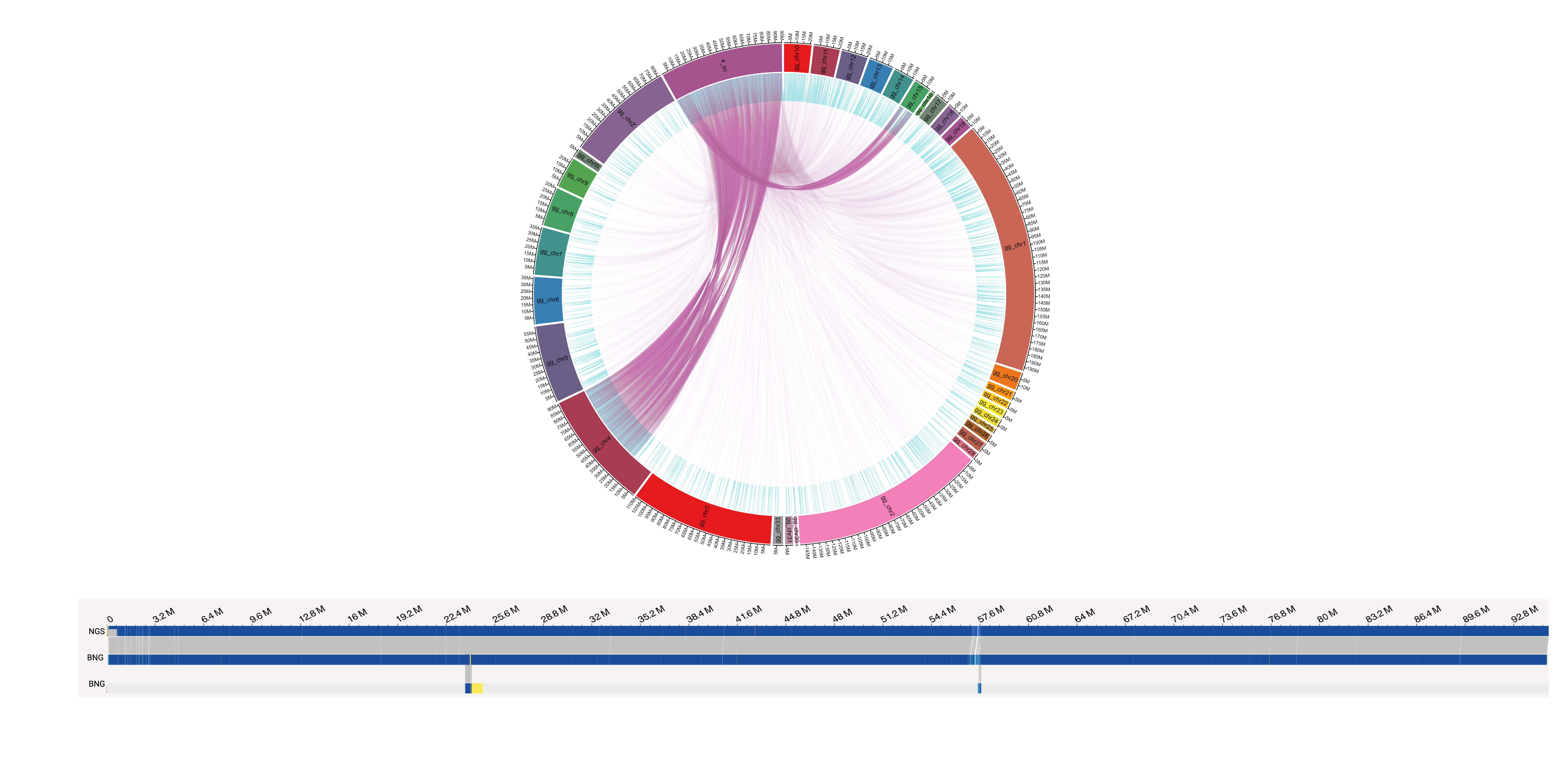

Supplement: jkad001_Supplementary_Data [file jkad001_supplementary_data.zip › Supplemental_Figure_10_G3-2022-403823.tif]

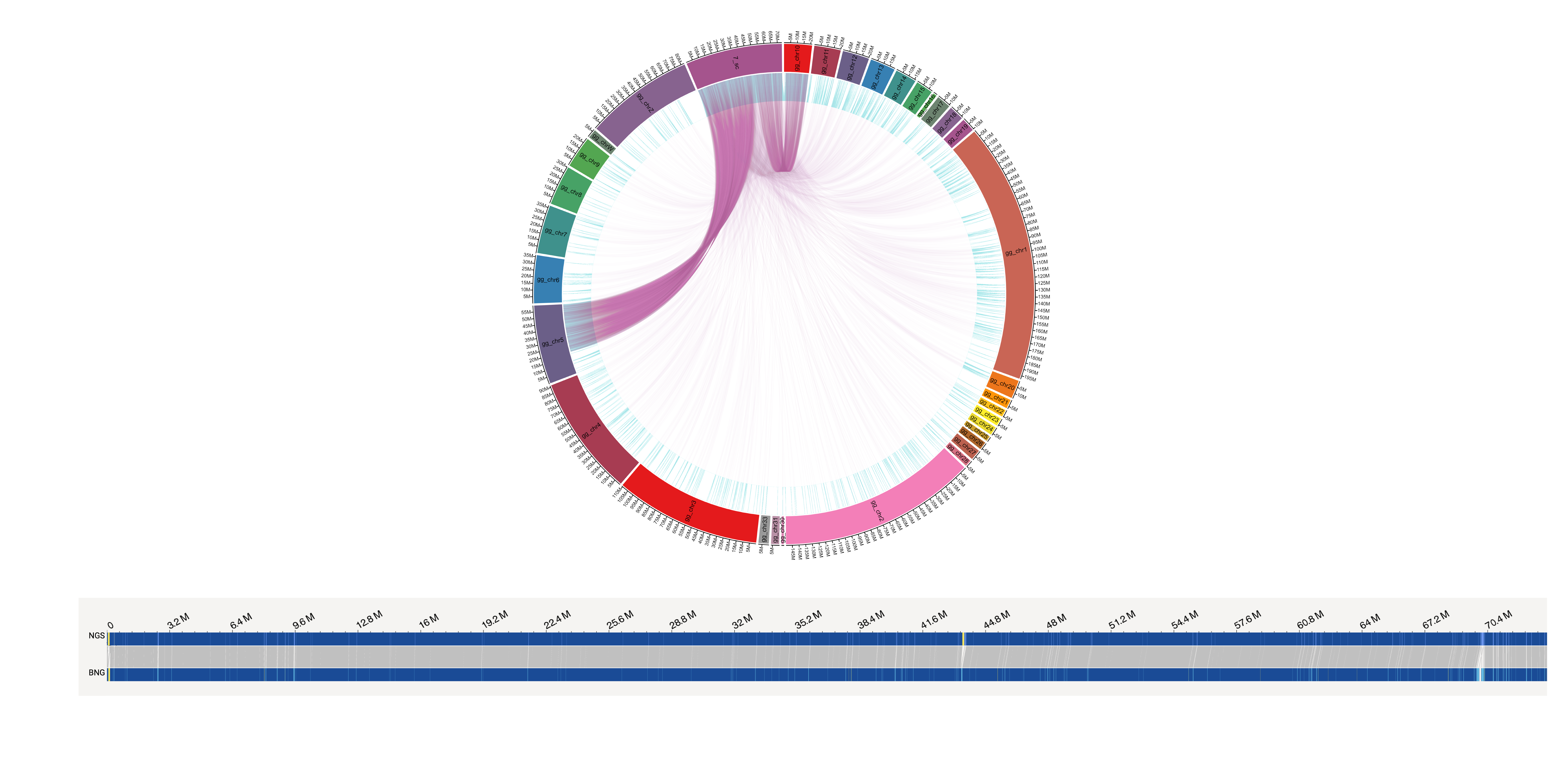

Supplement: jkad001_Supplementary_Data [file jkad001_supplementary_data.zip › Supplemental_Figure_11_G3-2022-403823.tif]

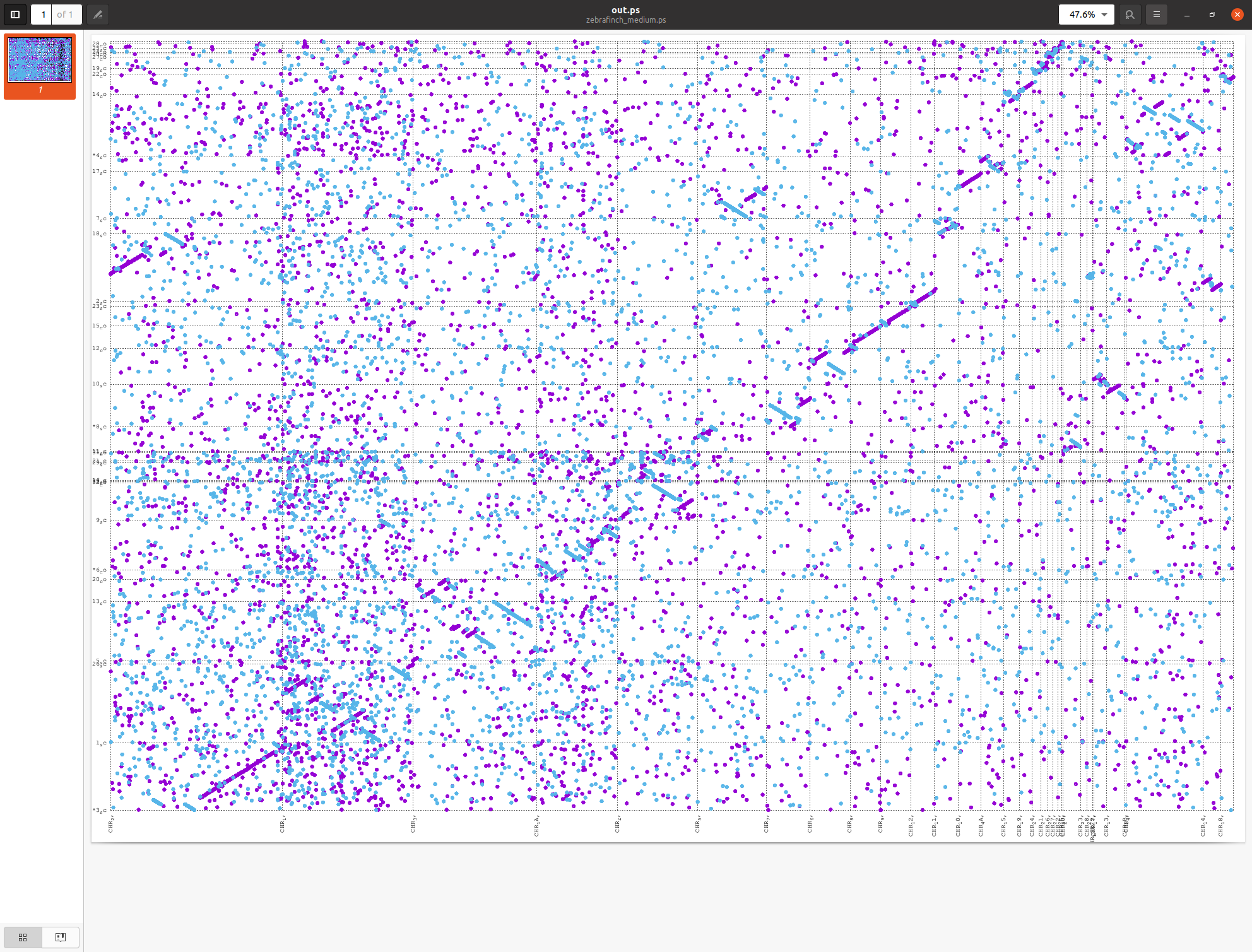

Supplement: jkad001_Supplementary_Data [file jkad001_supplementary_data.zip › Supplemental_Figure_2_G3-2022-403823.tif]

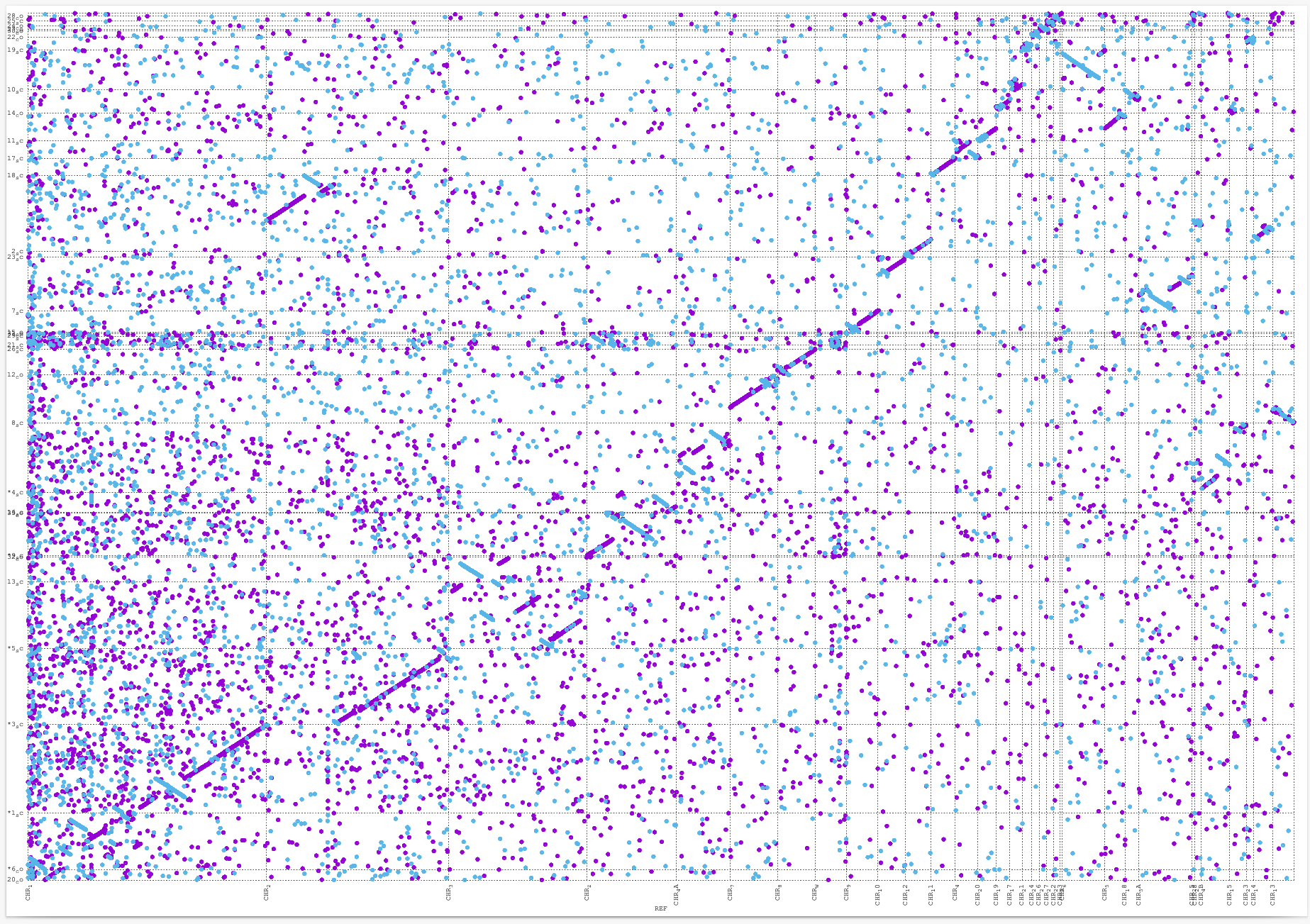

Supplement: jkad001_Supplementary_Data [file jkad001_supplementary_data.zip › Supplemental_Figure_3_G3-2022-403823.tif]

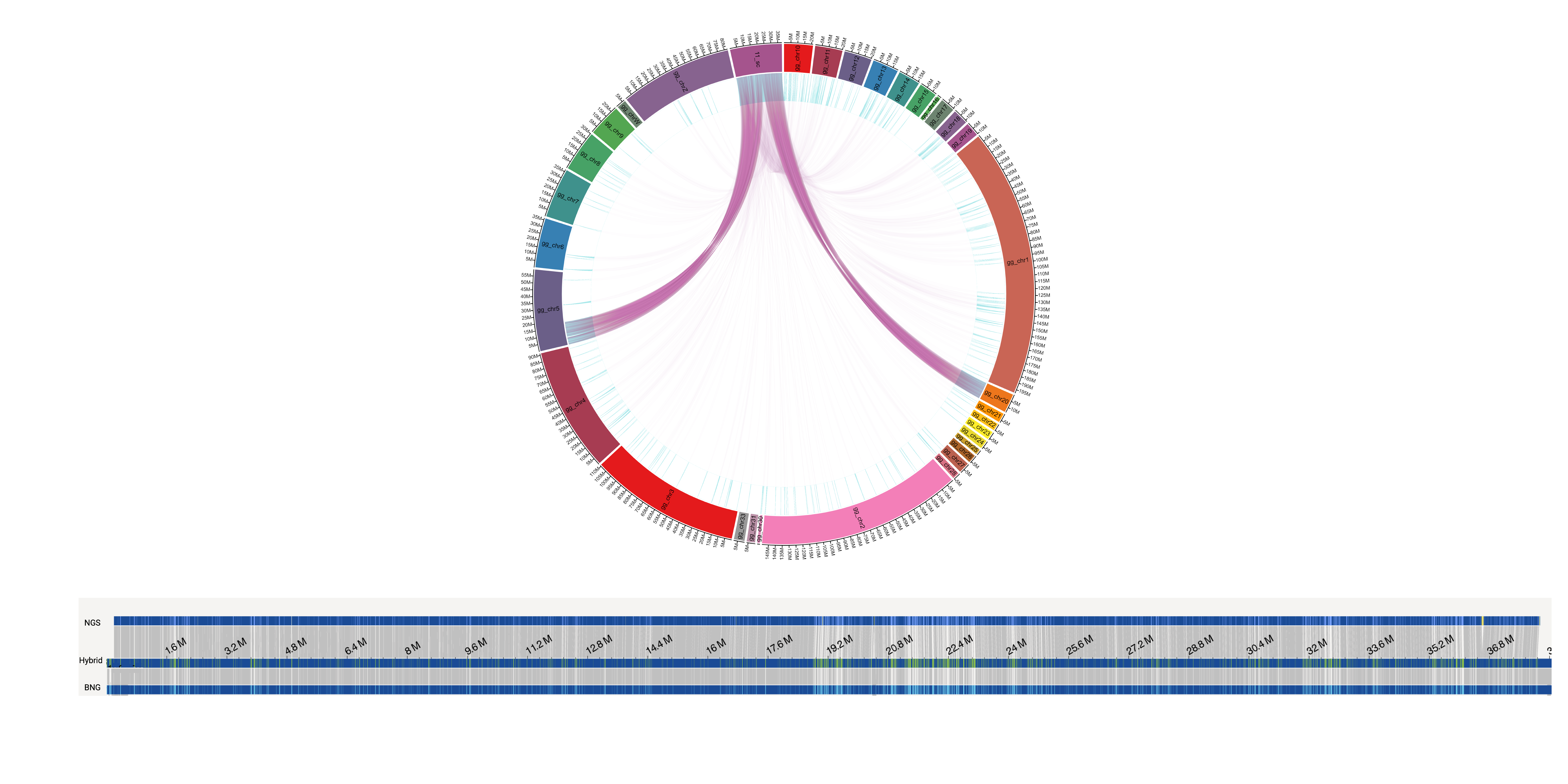

Supplement: jkad001_Supplementary_Data [file jkad001_supplementary_data.zip › Supplemental_Figure_4_G3-2022-403823.tif]

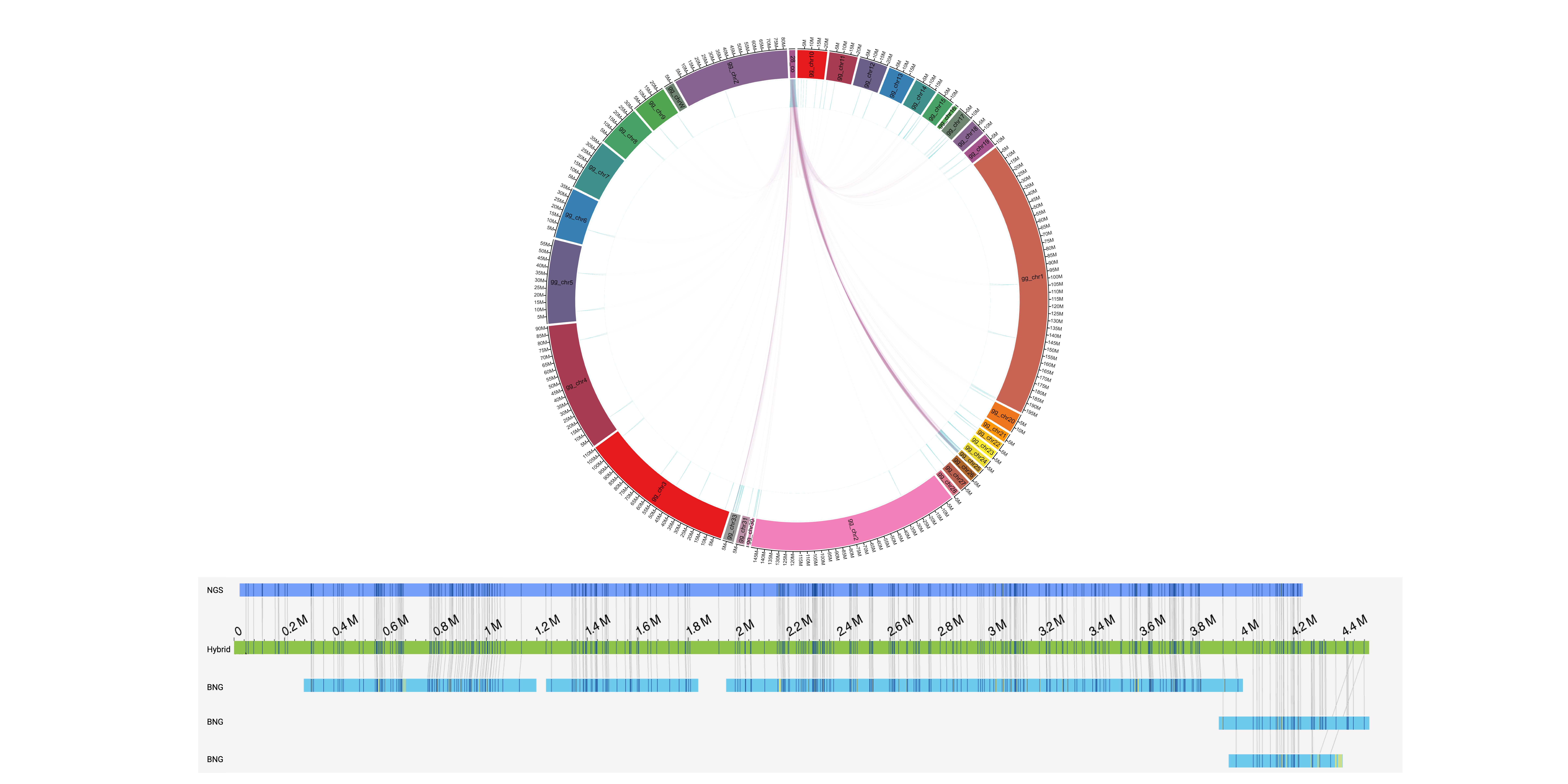

Supplement: jkad001_Supplementary_Data [file jkad001_supplementary_data.zip › Supplemental_Figure_5_G3-2022-403823.tif]

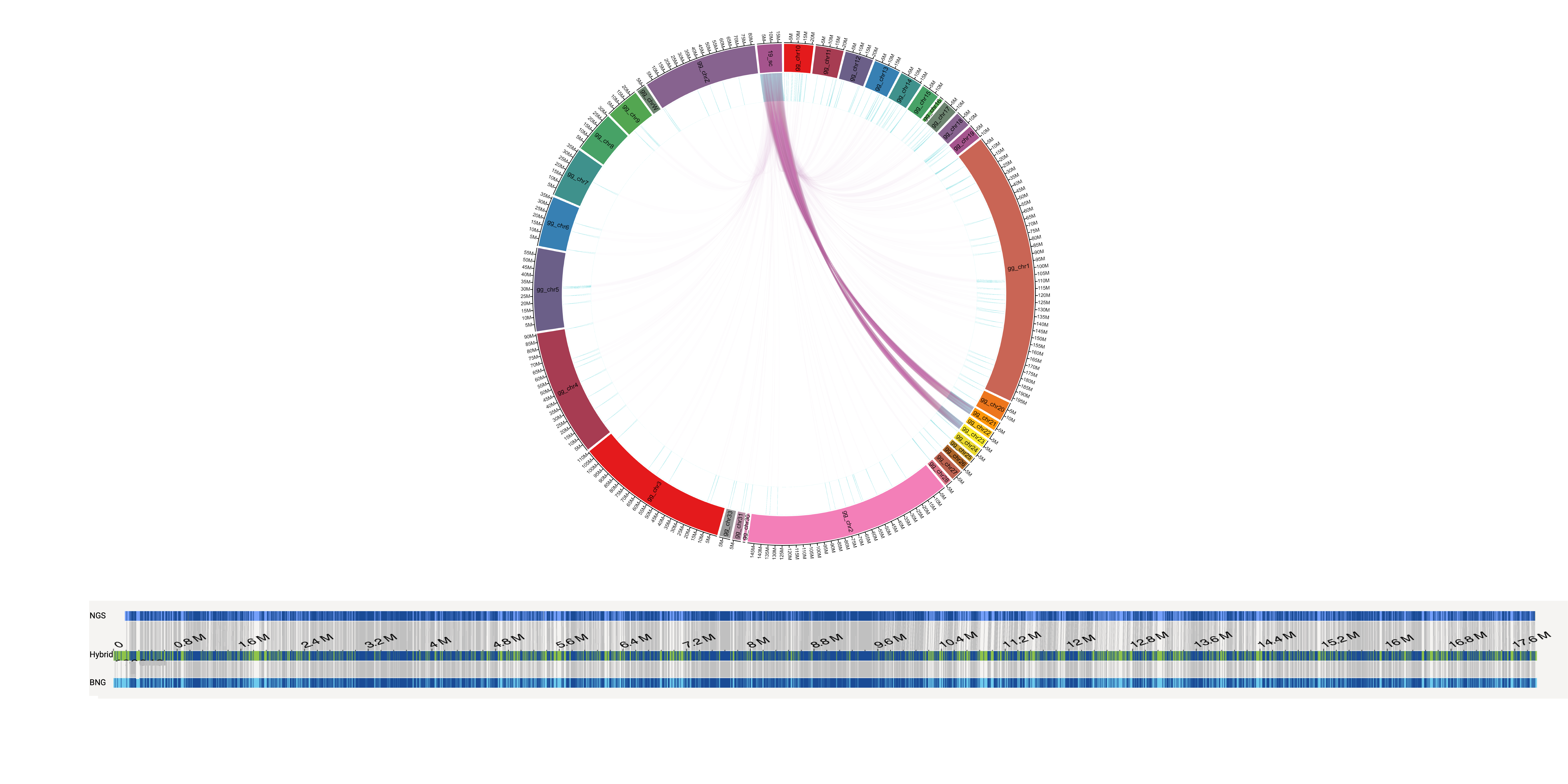

Supplement: jkad001_Supplementary_Data [file jkad001_supplementary_data.zip › Supplemental_Figure_6_G3-2022-403823.tif]

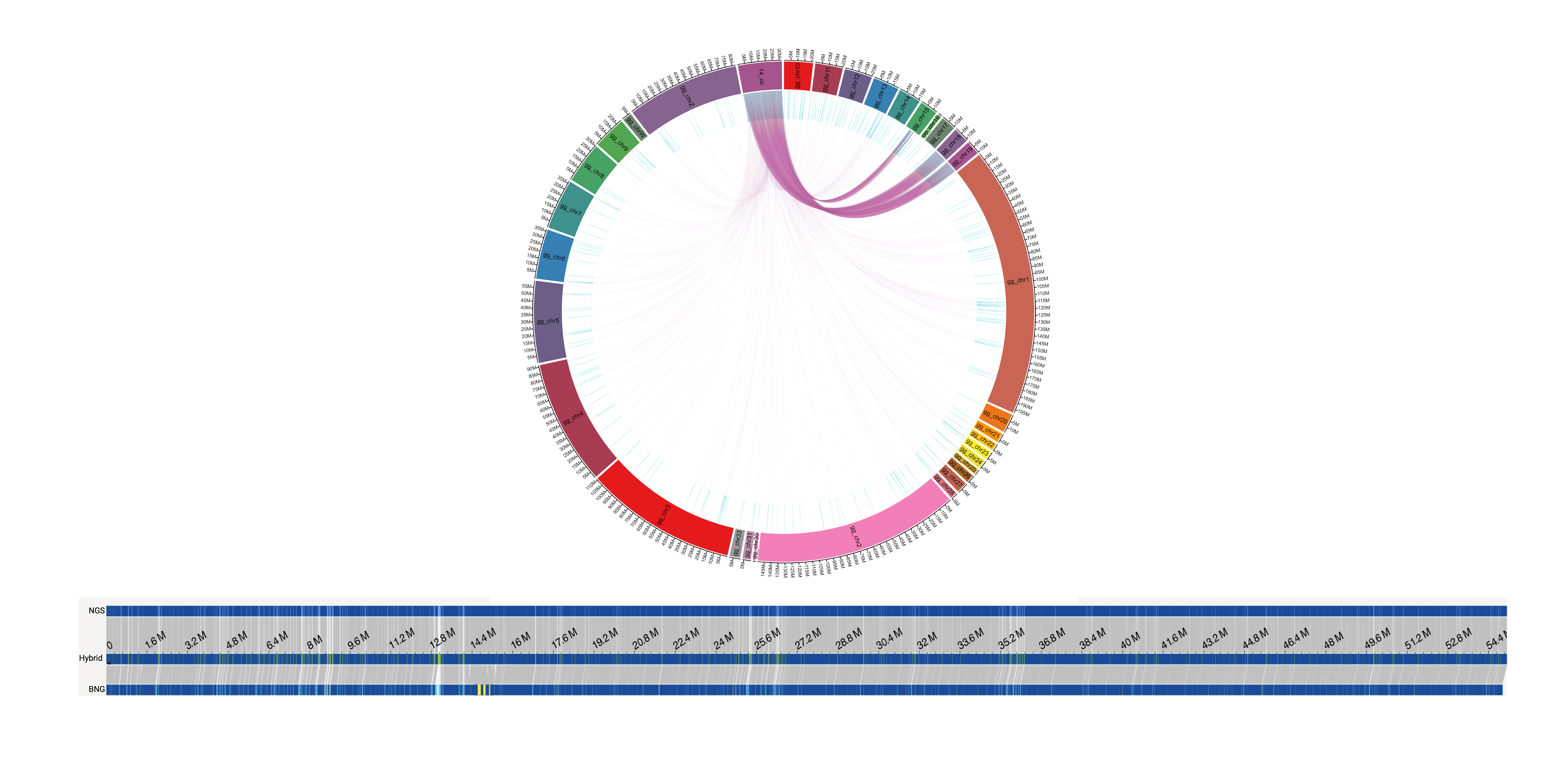

Supplement: jkad001_Supplementary_Data [file jkad001_supplementary_data.zip › Supplemental_Figure_7_G3-2022-403823.tif]

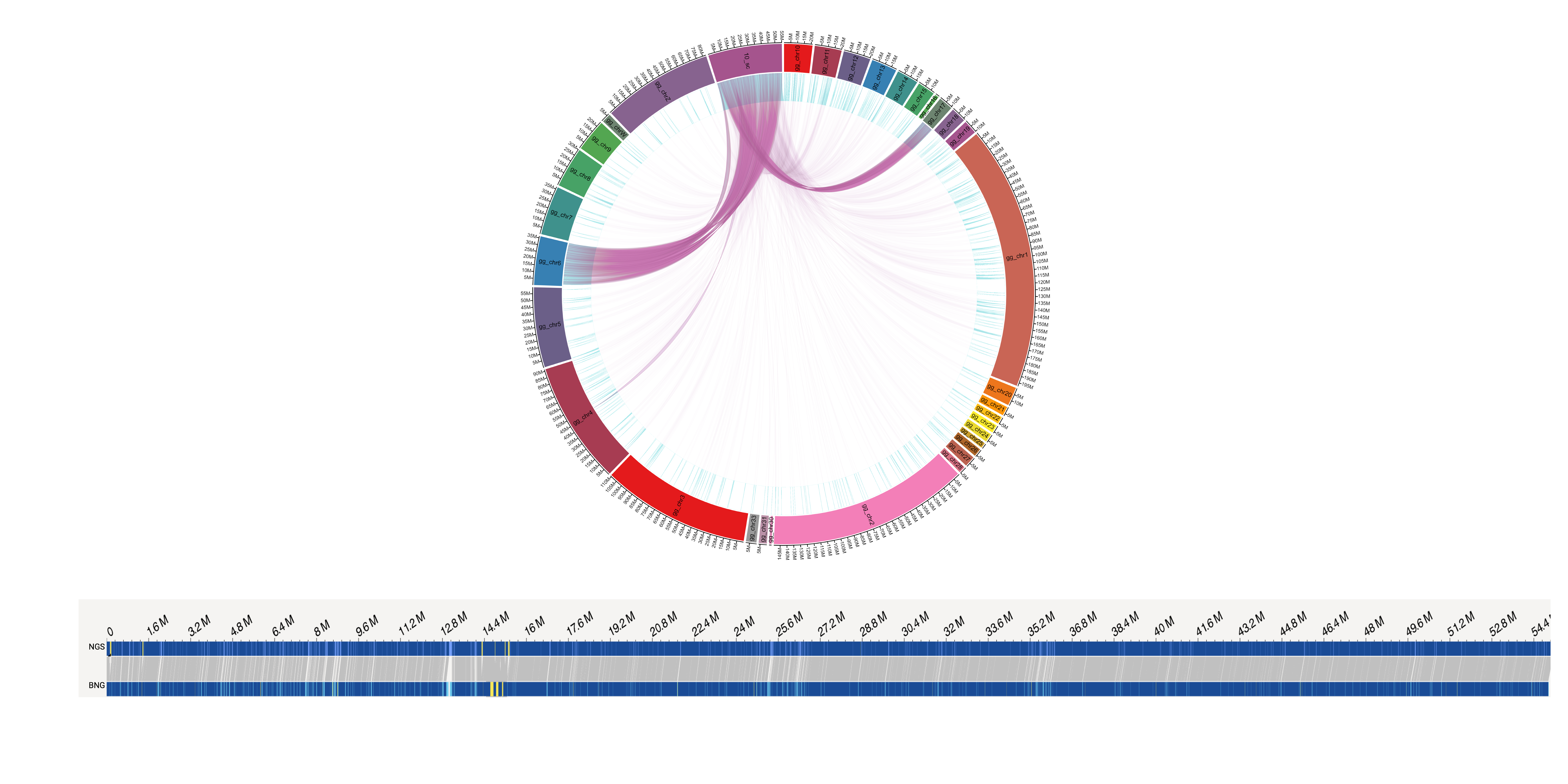

Supplement: jkad001_Supplementary_Data [file jkad001_supplementary_data.zip › Supplemental_Figure_8_G3-2022-403823.tif]

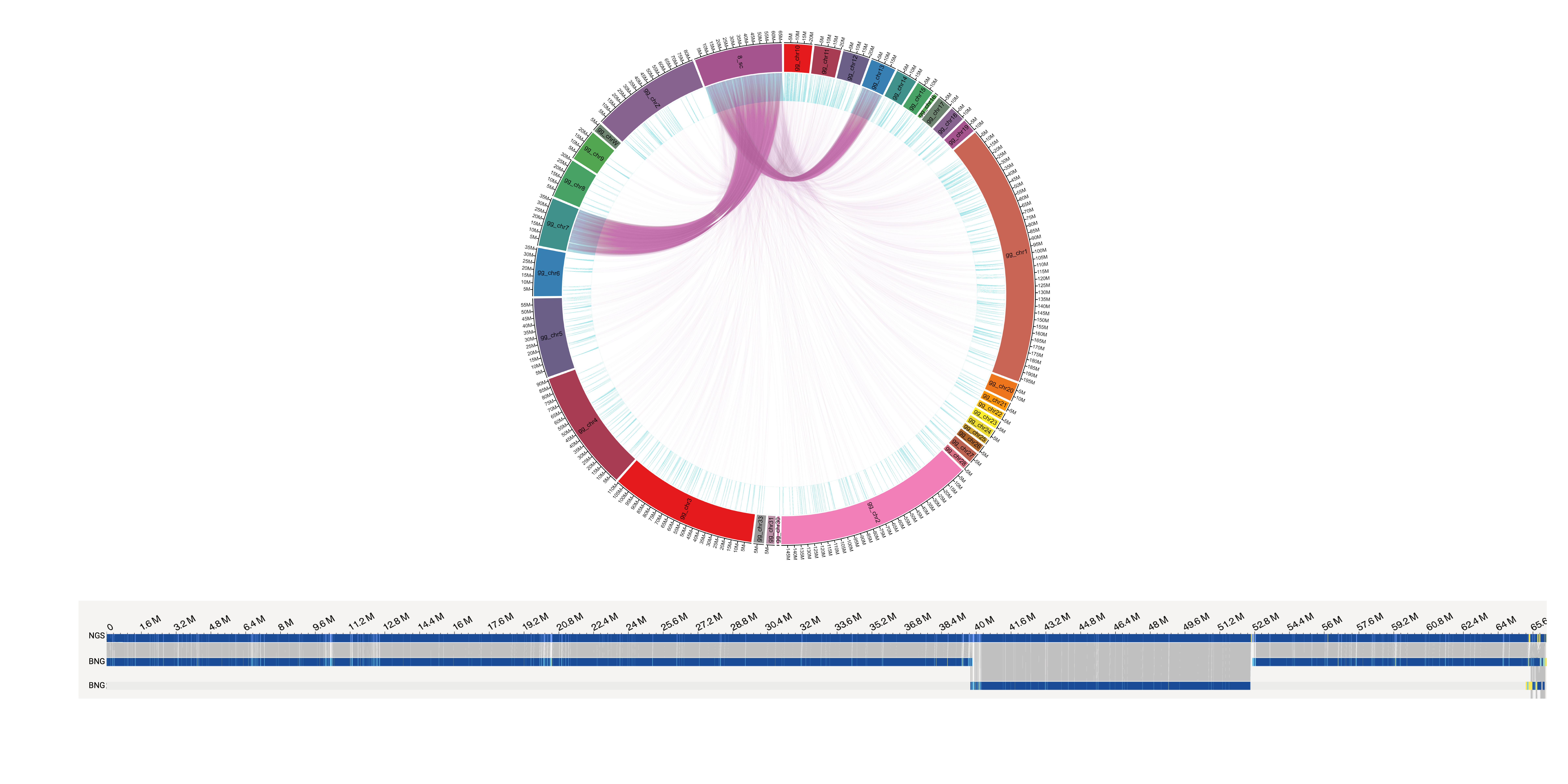

Supplement: jkad001_Supplementary_Data [file jkad001_supplementary_data.zip › Supplemental_Figure_9_G3-2022-403823.tif]
