# Supplementary material for: The gyrfalcon (Falco rusticolus) genome
Source: G3 (Bethesda). 2023 Jan 5;13(3):jkad001. doi: 10.1093/g3journal/jkad001 (PMC9997569; doi:10.1093/g3journal/jkad001)
Supplement: jkad001_Supplementary_Data [file jkad001_supplementary_data.zip › Supplemental_Material_Legends_G3-2022-403823.doc]

**SUPPLEMENTARY DATA**

Supplementary Figure 1. Dot plot of the *Falcon rusticolus* genome assembly (y-axis) versus the *Gallus gallus* genome assembly (x-axis)

Supplementary Figure 2. Dot plot of the *Falcon rusticolus* genome assembly (y-axis) versus the zebra finch genome assembly (x-axis)

Supplementary Figure 3. Dot plot of the *Falcon rusticolus* genome assembly (y-axis) versus the hummingbird genome assembly (x-axis)

Supplementary Figure 4. Circa plot comparing gyrfalcon 11_sc versus the entire set of domestic chicken chromosomes (specified as “gg_chromosome number”). Regions showing significant similarity are connected by violet lines. B) Bionano optical map validation for 11_sc. NGS: assembled sequence; BNG: Bionano map, Hybrid: Hybrid scaffold

Supplementary Figure 5. Circa plot comparing gyrfalcon 28_co with the entire set of domestic chicken chromosomes (specified as “gg_chromosome number”). Regions showing significant similarity are connected by violet lines. B) Bionano optical map validation for 28_co NGS: assembled sequence; BNG: Bionano map, Hybrid: Hybrid scaffold

Supplementary Figure 6. Circa plot comparing gyrfalcon 19_sc with the entire set of domestic chicken chromosomes (specified as “gg_chromosome number”). Regions showing significant similarity are connected by violet lines. B) Bionano optical map validation for 19_sc NGS: assembled sequence; BNG: Bionano map, Hybrid: Hybrid scaffold

Supplementary Figure 7. Circa plot comparing gyrfalcon 14_co with the entire set of domestic chicken chromosomes (specified as “gg_chromosome number”). Regions showing significant similarity are connected by violet lines. B) Bionano optical map validation for 14_co NGS: assembled sequence; BNG: Bionano map, Hybrid: Hybrid scaffold

Supplementary Figure 8. Circa plot comparing gyrfalcon 10_sc with the entire set of domestic chicken chromosomes (specified as “gg_chromosome number”). Regions showing significant similarity are connected by violet lines. B) Bionano optical map validation for 10_sc. NGS: assembled sequence; BNG: Bionano map

Supplementary Figure 9. Circa plot comparing gyrfalcon 8_sc with the entire set of domestic chicken chromosomes (specified as “gg_chromosome number”). Regions showing significant similarity are connected by violet lines. B) Bionano optical map validation for 8_scNGS: assembled sequence; BNG: Bionano map

Supplementary Figure 10. Circa plot comparing gyrfalcon 4_sc with the entire set of domestic chicken chromosomes (specified as “gg_chromosome number”). Regions showing significant similarity are connected by violet lines. B) Bionano optical map validation for 4_sc NGS: assembled sequence; BNG: Bionano map

Supplementary Figure 11. Circa plot comparing gyrfalcon 7_sc with the entire set of domestic chicken chromosomes (specified as “gg_chromosome number”). Regions showing significant similarity are connected by violet lines. B) Bionano optical map validation for 7_sc NGS: assembled sequence; BNG: Bionano map

Supplementary file 1: description of the locations and structures of the predicted genes

Supplementary file 2: multifasta file containing the TE library used in this work (excluding entries from Repbase)
